# Outward bulging of the right parietal bone in connection with fibrous dysplasia in an infant: a case report

**DOI:** 10.1186/1757-1626-1-347

**Published:** 2008-11-24

**Authors:** Ali Al Kaissi, Klaus Klaushofer, Franz Grill

**Affiliations:** 1 Ludwig Boltzmann Institute of Osteology, Hanusch Hospital of WGKK, Vienna, Austria; 2 AUVA Trauma Centre Meidling, 4th Medical Department, Hanusch Hospital, Vienna, Austria; 3 Orthopaedic Hospital of Speising, Paediatric Department, Vienna, Austria

## Abstract

**Background:**

Fibrous dysplasia (FD) is a developmental disease of bone in which there is replacement of normal spongiosa and filling of the medullary cavity of affected bones by an abnormal fibrous tissue that contains trabeculae of poorly calcified primitive bone formed by osseous metaplasia. Fibrous dysplasia is a common benign bone disease existing in monostotic and polyostotic forms. It is sometimes associated with aneurysmal bone cysts, and it is a component of McCune-Albright and Mazabraud syndromes.

**Case presentation:**

We describe here a 4-months old Austrian infant who presented with a hard bulging painless mass of (5 x 3 cm) of the right parietal bone. Radiographs showed a large irregular osteolytic lesion. T1-weighted MR image showed significant expansile lesion associated with a dense zone of calcification in the diploic space. To the best of our knowledge this is the first clinical report of an infant with early presentation of monostotic fibrous dysplasia of the right parietal bone.

**Conclusion:**

Fibrous dysplasia of the skull is a painless progressively expanding destructive bone swellings produce cosmetic deformities. The clinical course may be unpredictable, with sudden appearance of symptoms, some of which can be important and irreversible. In our present patient, the possibility that an early surgical correction might positively interfere with the natural history of the lesion has to be evaluated by taking into account the obvious difficulties that will be encountered in reconstructing the skull after a wide excision of the pathologic bone.

## Background

Skeletal fibrous dysplasia is a localised disorder of bone characterised by abnormal proliferation of fibrous tissue interspersed with normal or immature bone, endocrine dysfunction, abnormal pigmentation, and precocious puberty in girls. Fibrous dysplasia, when it occurs in the craniofacial region, mostly involves the skull base and is rarely localized in the cranial vault. FD is primarily a developmental abnormality of the bone forming mesenchyme in which fibrous tissue gradually expands and replaces bone. It is believed to be a non-neoplastic hamartomatous developmental lesion of unknown etiology.

Monostotic fibrous dysplasia frequently shows lesional distribution in the ribs, jaw, skull, or long bones. The clinical presentation of monostotic fibrous dysplasia is usually painless and asymptomatic, and it is often discovered accidentally on radiographic film. Polyostotic fibrous dysplasia may be an indication of Albright's syndrome [1-3].

Uncomplicated monostotic lesions are asymptomatic and do not cause significant deformity. As a rule, they do not convert to the polyostotic form, do not increase in size and become inactive at puberty. The polyostotic variety is more severe with the involvement of multiple sites. It results in significant deformities and, although the lesions become quiescent after puberty, the deformities may progress. The true incidence and prevalence of fibrous dysplasia are difficult to estimate, but the lesions are not rare. They are reported to represent approximately 5–7% of benign bone tumours. The lesions of FD develop during skeletal formation and growth and have a variable natural evolution. Clinical presentation may occur at any age, with the majority of lesions being detected by the age of 30 years. The disease has no gender predilection. The differential diagnosis of fibrous dysplasia includes meningioma, aneurysmal bone cyst, unicameral cyst, ossifying or non-ossifying fibroma, Paget's disease, osteochondroma, eosinophilic granuloma, calcified cephalohematoma and sarcomatous neoplasm [1-7].

## Case presentation

**Figure 1:**
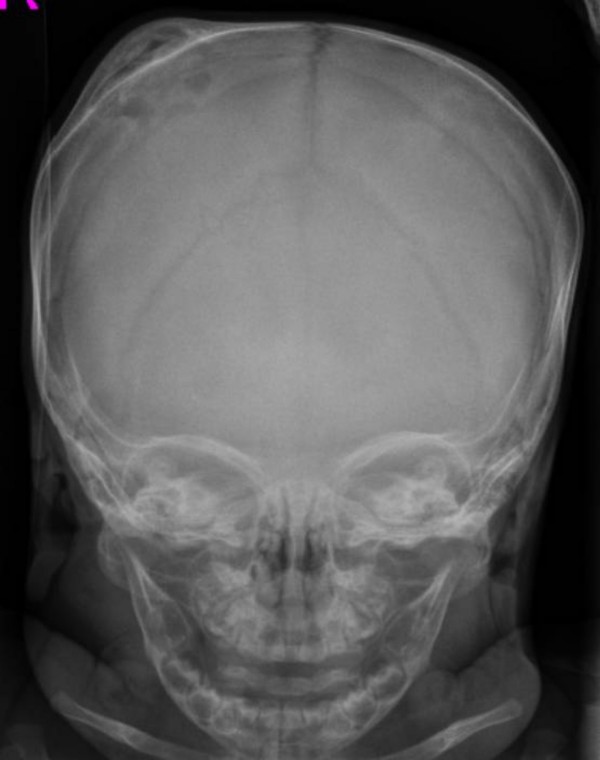
Anteroposterior skull radiographs showed a multicystic lesion with slightly sclerotic margin in the right parietal bone, associated with significant outward bulging of the outer table without destruction of the inner table in tangential view of the right parietal bone.

**Figure 2:**
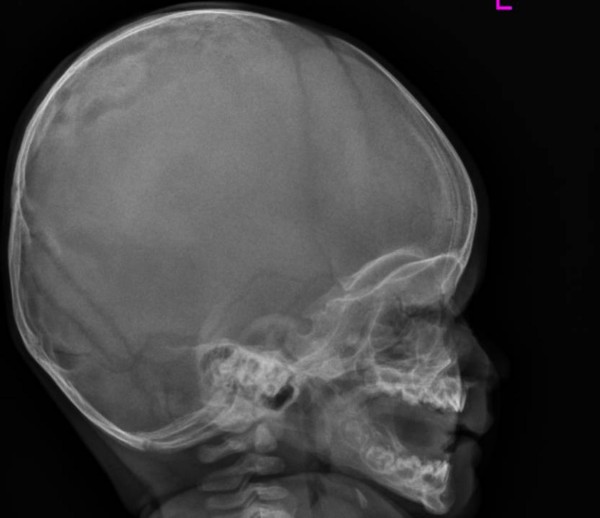
Lateral radiograph of the skull showed large osteolytic/multicystic lesion associated with thick sclerotic rind in the right parietal bone.

**Figure 3:**
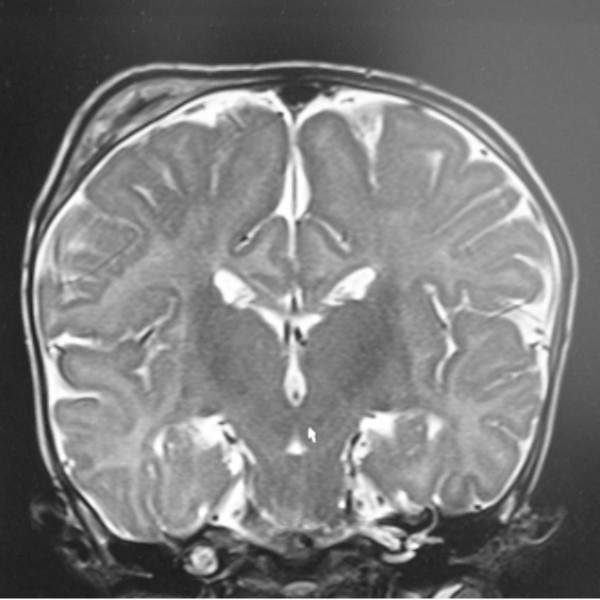
**T_1 _weighted image of the skull showed expansile lesion within the parietal bone**. The lesion is larger than appreciated on the radiograph. There is mixed hyper and hypointenstities. It is significantly expanded with well-defined margins.

**Figure 4:**
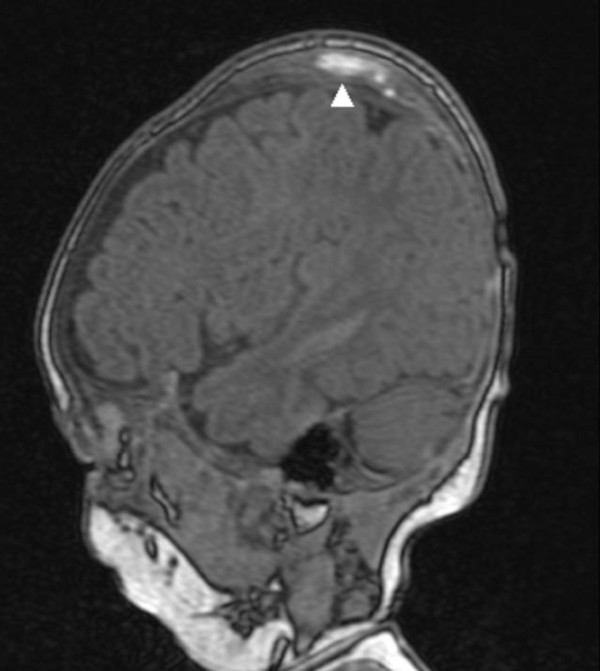
Sagittal T1-weighted MR image showed significant expansile lesion associated with a dense zone of calcification within the mass-arrow (fig 4).

A 4-months old male infant was referred to our department because of progressive/painless bulging over the right parietal bone. He was the first child to non-consanguineous parents. The mother was a 28-year gravida 1 abortus 0 married to a 31-year-old unrelated man. He was a product of uneventful gestation. At birth his weight, length and head circumference were around the 50 th percentile. Recently, his parents noticed a hard/painless mass over his right parietal bone. Clinical examination showed a 4-months old child with no associated dysmorphic facial features or any associated craniofacial disfigurement, apart from a hard mass over the right parietal bone. Hearing and vision were normal. Neurological examination was normal. Skin showed no peculiar stigmata. Anteroposterior skull radiographs showed a multicystic lesion with slightly sclerotic margin in the right parietal bone, associated with significant outward bulging of the outer table without destruction of the inner table in tangential view of the right parietal bone (fig 1). Lateral radiograph of the skull showed large osteolytic/multicystic lesion associated with thick sclerotic rind in the right parietal bone (fig 2). T_1 _weighted image of the skull showed expansile lesion within the parietal bone. The lesion is larger than appreciated on the radiograph. There is mixed hyper and hypointenstities. It is significantly expanded with well-defined margins (fig 3). Sagittal T1-weighted MR image showed significant expansile lesion associated with a dense zone of calcification within the mass-arrow (fig 4). Skeletal survey of the child showed no other bones involved. The child underwent a series of investigations, included complete blood cell counts, urine biochemistry, chromosomal analysis and metabolic tests, which aimed to test calcium, phosphorous, and vitamin D metabolism, all were normal. Hormonal investigations included thyroid hormones were normal.

## Discussion

Lichtenstein introduced the term fibrous dysplasia for the first time, although the condition had been known previously by a variety of names such as osteitis fibrosa, osteodystrophia fibrosa, fibrous osteoma, ossifying fibroma, unilateral von Recklinghausen's disease, and many others [1].

Fibrous dysplasia might be mono-ostotic, poly-ostotic, involve a large area of the skull or involve single bones. The lesion of fibrous dysplasia appears in three distinctive clinical patterns. The most severe form of FD is McCune-Albright syndrome, which is more commonly found in females and is associated with short stature due to premature closure of the epiphyses and with endocrine abnormalities and pigmented cutaneous lesions [2]. Another severe form is Mazabraud syndrome. It is characterised by the association of polyostotic fibrous dysplasia of the bones with solitary tumours of large muscle groups, occurring predominantly in the lower limbs, and myxomas [3].

FD is primarily a developmental abnormality of the bone-forming mesenchyme in which fibrous tissue gradually expands and replaces the bone. It is believed to be a non-neoplastic hamartomatous developmental lesion of bone of unknown origin. Fibrous dysplasia is a genetic disease caused by postzygotic, activating mutations of the GNAS gene, which impair the GTPase activity of Gs-alpha thus resulting in excess intracellular cAMP. In most cases the radiographic characteristics of polyostotic FD and the clinical information are sufficient to allow the practitioner to make a diagnosis without a biopsy [4-7]

Fibrous dysplasia of the skull bones is usually a benign condition, which begins in childhood and stabilises at puberty. About a third of patients have further progression in adulthood. It might be mono-ostotic, poly-ostotic, involve a large area of the skull or involve single bones such as the sphenoid ridge. When the sphenoid ridge is involved, the commonest presenting sign is proptosis. There might be two forms, the sclerotic and lytic forms. A wide nasal bridge and nasal obstruction can also occur [8-10]. Lee et al., [11] studied 38 patients with fibrous dysplasia of the lesser wing of the sphenoid bone. They concluded that encasement of the optic canal in fibrous dysplasia causes narrowing of the canal but this in itself does not result in visual loss. Prophylactic decompression of the optic nerve does not appear to be indicated on the basis of the presence of fibrous dysplasia on diagnostic images alone.

The European Paediatric Orthopaedic Society has performed a multicenter clinicopathologic study to gain insight into the natural history of fibrous dysplasia. Fifty-three patients from eleven centres were included. Twenty-three patients with a mean age of fifteen years, had monostotic involvements. Ten, with a mean age of eleven years, had polyostotic involvement, and twenty with a mean age of 4.5 years, had McCune-Albright syndrome. In the cohort with monostotic disease, the most common site of involvement was the femur. Lesions in that group presented as an incidental finding or with pain, swelling or fracture. The other common areas of involvement were the tibia, humerus, rib, clavicle, and craniofacial skeleton. A majority of the monostotic cases did not progress, and the long-term outcome was usually satisfactory in those cases regardless of treatment [12].

## Conclusion

Most lesions of FD are monostotic, asymptomatic, and identified incidentally and can be treated with clinical observation and patient education. Knowledge of the various appearances, complications, and associations of fibrous dysplasia is important to ensure the accurate diagnosis and appropriate management of this disease. Surgery is indicated for confirmatory biopsy, correction of deformity, and prevention of pathologic fracture and or eradication of symptomatic lesions. It has been postulated, however, that surgical treatment is needed when the patient have significant clinical symptoms.

## Abbreviations

FD: Fibrous dysplasia; GNAS 1: guanine nucleotide-binding protein, α-stimulating activity polypeptide 1.

## Competing interests

The authors declare that they have no competing interests.

## Authors' contributions

All of the authors were involved in the clinico-radiographic assessment and finalising the paper. All authors have read and approved the final version of the paper.

## Consent

Written informed consent was obtained from the parents for the purpose of publication of the manuscript and figures of their child. A copy of the written consent is available for review by the editor-in-Chief of this journal.
